# Self Care Behavior among Patients with Diabetes in Harari, Eastern Ethiopia: The Health Belief Model Perspective

**DOI:** 10.1371/journal.pone.0035515

**Published:** 2012-04-17

**Authors:** Ketema Ayele, Bisrat Tesfa, Lakew Abebe, Tizta Tilahun, Eshetu Girma

**Affiliations:** 1 Harar Health Science College, Harai, Ethiopia; 2 Department of Health Education and Behavioral Sciences, Jimma University, Jimma, Ethiopia; 3 Department of Population and Family Health, Jimma University, Jimma, Ethiopia; University of Sao Paulo, Brazil

## Abstract

**Background:**

Diabetes mellitus is a chronic disease that requires lifelong medical treatments and a life style adjustment. To prevent serious morbidity and mortality, it requires dedication to demanding self-care behaviors in multiple domains. The objective of this study was to identify predictors of self care behaviors among patients with diabetes.

**Methods:**

From a total of 425 follow up diabetic patients, a quantitative cross sectional study was conducted among 222 of them from three different hospitals in Harar town, from March to April, 2011. The sample was taken using simple random sampling method. Data was collected using pretested questionnaire. Descriptive statistics multiple logistic regression analysis were also used to assess the predicators of self care behaviors among patients with diabetes.

**Result:**

Majority of the study respondents 134 (60.4%) were female and the mean age was 49.7 (SD±14.7) years. More than half 147(66.2%) of them were medically diagnosed with type-2 diabetes. 208(93.7%) had general knowledge about diabetes and specific knowledge about diabetes self care 207(93.2%). Large proportion of them had moderate perceived susceptibility 174(78.4%) and severity 112(50.5%). More than half of the respondents 149(67.1%) had less perceived barrier while only 30 (13.5%) of them had high self efficacy to self care practices related to diabetes mellitus. Only 87(39.2%) followed the recommended self care practices on diabetes.

**Conclusions:**

Patients with less frequent information were less likely to take diabetes self care. Patients who were more educated, middle income, had high perceived severity of diabetes and less perceived barrier to self care were more likely to take diabetes self care. To increase the self care behavior, diabetes messages should focus on severity of diabetes and how to overcome barriers for self care by segmenting the audiences based on income and educational status with increasing the frequency and reach of message on diabetes.

## Introduction

Diabetes-related complications are major causes of morbidity and mortality; and have significant impact on the patients' quality of life and productivity. By 2025, the worldwide prevalence is projected to be 6.3 percent, a 24-percent increase over the 2003 rate [1]. The prevalence of diabetes mellitus is increasing in developing countries due to population growth, aging, unhealthy diets, obesity and sedentary lifestyles [2]. In the developed countries the prevalence of diabetes is reaching up to 16 percent [3]. It has been predicted that in year 2025 the number of people with diabetes will have been doubled. During that time out of those 300 million people with diabetes, 76% will be living in low-income countries [2].

In Ethiopia the prevalence of non-communicable diseases, including hypertension, cardiovascular diseases and diabetes mellitus is increasing with changes in people's lifestyles [4]. Hospital based studies in Addis Ababa showed that the prevalence of diabetes has increased from 1.9 percent in 1970 to 9.5 percent in 1999 [3]. The overall prevalence of diabetes in the northern Ethiopia has been reported as approximately 0.3 percent and a mortality of 31 percent of people with Type 1 diabetes has been described in Addis Ababa [5]. Even though the actual number was not known, World health organization has estimated the number of diabetic cases in Ethiopia to be 800,000 by the year 2000, and the number is expected to increase to 1.8 million by 2030 [6].

To prevent serious morbidity and mortality, diabetes treatment requires dedication to demanding self-care behaviors in multiple domains, including food choices, physical activity, proper medications intake and blood glucose monitoring [7]. A study showed that older adults were very compliant with taking medication but were only moderately compliant to diet and self-glucose monitoring and least compliant to exercise. Finding on the same study showed that 60 percent of the study respondents reported that understanding their diet was a barrier for self care [8]. Furthermore other study has shown that poor glycemic control is correlated with longer duration of being diabetic and the occurrence of late complications [9].

Finding from a study indicated that patient reports of self care behavior did not differ substantially by type of diabetes; whereas, providers reported significantly better adherence among their patients with Type 1 diabetes than those with Type 2 diabetes for most regimen domains [8]. Though, there was significant variation across countries, self care behavior on diabetes is less than optimal in all countries. Only 46 percent of Type 1 patients and 39 percent of Type 2 patients achieved complete success in at least two-thirds of their self-care domains. Methods for generating patient and provider estimates of self care differed; provider reported success in self care was much lower than patient reported success for both Type 1 and Type 2 diabetes [8].

Another study conducted on psychosocial problems and barriers to diabetes self care in different countries showed that Patient-reported success was significantly higher for medication and appointment keeping than for diet and exercise [7]. A study on self care activities showed that, the mean number of days per week that patients exhibited good diet behavior was 3.16 while the mean days per week on which patients undertook daily exercise was 3.34. The majority of patients 82 percent were considered to have poor diet behaviors while 52 percent of the patients were found to have poor exercise behaviors [10].

The main treatments goal for patients with diabetes are to prevent or minimize the acute or chronic complications mainly by following the self care practices which includes regular exercise, taking recommended diet, proper intake of prescribed medications and blood glucose monitoring. Though self care behaviors are very determining factors to control the disease and its related complications, self care is highly challenging since factors such as diabetic patient's knowledge, physical skills, emotional factors, self efficacy and others perceptions of the patient interact and affects the self care behavior [11]. Generally, to perform the patient with the above mentioned practices the risk perceptions of the patient towards the disease and complications is the main determining factors. In Ethiopia, there was no previously documented study with regard to diabetes self care behavior. There for this study was aimed at identified personal perceptions of diabetes patients towards self-care behaviors. The findings will be relevant to identify important programmatic areas for interventions targeted at minimizing complications due to diabetes and can serve as a baseline for further researches.

We used the Health Belief Model as a conceptual model for understanding and predicting adherence to self care behavior. We utilized the model with respect to diabetes to its essential hypothesis that self care increases as a function of the patient's perceptions of greater susceptibility to the illness, greater disease severity, including related complications, more perceived benefits of self care (i.e. perception of individuals about the benefits of self care behaviors and its effectiveness), fewer perceived barrier to self care (i.e. individuals own evaluation of obstacles for self care behavior), more social cues or prompts to self care and greater self-efficacy to self care (i.e. Perception about individuals ability to perform self care behaviors) [12].

## Methods

Facility based cross-sectional study was conducted in diabetic follow-up clinics of three hospitals in Harari region between March and April 2011. Harari is a regional state found in the eastern part of Ethiopia. It is located 515 km away from Addis Ababa (the capital city). It has an estimated total population of 196,000, consisting of 100,000 men and 96,000 women.

The study was conducted among 222 adult diabetic follow up patients in three hospitals of Harar town (*Hiwot Fana, Jugel and police hospitals*). The sample size was determined using a single population proportion sample size estimation method by assuming that 50% of the patients follow the recommended self care behaviors (to obtain the maximum representative sample size since no similar study was found in the area) with 95% confidence interval. Finally we calculated 222 patients by using population correction formula. The three hospitals have a separate follow- up clinics for diabetes follow up services. The total adult diabetic follow-up patients in the three hospitals were 425. We included all the 425 previously registered patients in the sampling frame. Based on the total adult follow up diabetic patients in each hospital we allocated the sample to the three hospitals proportionally. Then we selected the study respondents with simple random sampling technique. The list of patients (sampling frame) was obtained from the registration books of each follow up clinics of the hospitals. Since each patient had at least one appointment within a month, we have waited a maximum of a month to get a selected study participant.

Data was collected by six trained nurses using face to face interview method. We used a questionnaire which was adopted from other studies with internal consistency of α = 0.87 [13], [14] in the latter study. The questionnaire was prepared in English then translated in to Amharic language (local language) and back translated in to English language by another person to check its semantic equivalence. It was also pretested in a military hospital. The military hospital was excluded since they are different from the residents in their socio-demographic characteristics and mobile in their nature. In addition to the socio-demographic data, the questionnaire included items about all the constructs of the health belief model (perceived susceptibility and severity to diabetes complications, perceived benefits and barriers to self care, self efficacy to self care, and cues to action on self care).

We measured diabetes self care using 10 items on physical exercise, diet, medication and blood glucose measurement. We classified the self care as ‘good self care practice’ and ‘not good self care practice’; respondents were labeled to have “good self care” if they scored above 50% of the total self care practices in the last three days. To measure, perceived [susceptibility (8 items), severity (4 items), benefit (7 items), barrier (12 items)] and self efficacy (10 items) were used. We labeled the respondents to have these constructs based on correct response of 8o% or more as high, 50%–79% as moderate and less than 50% as less of the above constructs.

We measured the general knowledge of respondents on diabetes and knowledge on self care on diabetes. Respondents were categorized as having good general knowledge on diabetes if they responded correctly to more than two responses out of four questions; less knowledgeable if they responded only one question correctly and not knowledgeable if they did not respond all the questions. Knowledge on diabetes self care was categorized as good self care knowledge (respondents who responded all the three correct answers among three questions), less knowledgeable (respondents reacted to one or two correct answers among the three questions) and not knowledgeable (respondents who responded incorrect answer for all questions) for diabetes self care knowledge questions. Weight and height of the patients were measured and BMI was calculated and was classified as underweight, normal weight, overweight and obese. Weight and height measurements were taken during their routine check up at the health care facilities. The type of diabetes was also taken from their registration book

After cleaning and editing the data analyzed using SPSS version 16.0. Descriptive statistics was used to determine mean and frequency of dependent and independent variables. Variables which show significant association on bivariate analyses were fitted in to multiple logistic regression model to determine the independent predicators for diabetes self care.

### Ethics statement

Jimma University ethical committee of college of Public Health and Medical Sciences approved this study. Verbal consent was obtained from each respondent and the ethical committee approved the procedure since the study was a survey and with no any harm to the respondents.

## Results

### Socio- demographic characteristics

A total of 222 diabetes patient were involved in this study. The response rate in this study was 100%. Among the total of 222 respondents, 88(39.6%) and 134 (60.4%) were males and females respectively. The mean age was 49.7 (SD±14.7) years. More than half of them 127(57.2%) were married and Large proportion was unable to read and write 94(42.4%). Majority of the respondents were house wife where 67(30.2%) and government employee 48(21.6%). The mean average monthly income was 660.5 (SD±506.8) ET Birr or 39.3 (30.2) USD (Table 1).

**Table 1:** Socio-demographic characteristics of patients with diabetes in Harari; Eastern Ethiopia, 2011.

Variables	Number (%)
**Gender**	
Female	134(60.4)
Male	88(39.6)
**Age**	
18–25	14(6.3)
26–35	33(14.9)
36–45	35(15.4)
46–55	58(26.1)
>55	82(36.9)
**Marital Statues**	
Single	55(24.8)
Married	127(57.2)
Divorced	33(14.9)
Widowed	7(3.2)
**Religion**	
Orthodox	107(48.2)
Muslim	98(44.1)
Protestant	17(7.7)
**Living Conditions**	
With Family	196 (88.3)
Alone	26(11.7)
**Ethnicity**	
Amhara	100(45.6)
Oromo	81(36.5)
Harari	23(10.4)
Tigere	7(3.2)
Gurage	6(2.7)
Others*	5 (2.3)
**Educational Status**	
Unable to read and write	94 (42.4)
Below 6–12 grade	35(15.8)
From 6–12 grade	60(27.6)
Above 12 grade	33(14.9)
**Average monthly Income**	
Low	64 (28.8)
Middle	77(34.7)
High	36(16.2)
Very high	29(13.1)

* Others: - Afar, Somali.

### Background information and cues to action

One hundred forty seven (66.2%) of the respondents were medically diagnosed with type-2 diabetes. The mean duration since medically diagnosed for diabetes was 1.74 (SD±0.9) years. One hundred sixty two (73.0%) measured their blood glucose level once in a month. The mean BMI was 23.8(SD±3.9) and most of them 136(61.3%) were found in the normal weight category, but 13(5.9%) were obese. One hundred seventy four (78.4%) heard about diabetes self care in the last one month; among these 97(43.7%) of them heard from more than one source. The main source of information was health professionals 67(30.2%) (Table 2).

**Table 2:** Background characteristics of patients with diabetes in Harari; Eastern Ethiopia, 2011.

Variables	Number (%)
**Type of diabetes**	
Type 2 DM	147 (66.2)
Type 1 DM	75(33.8)
**Frequency of blood sugar measurement**	
Monthly	162(73.0)
Once a week	42(8.2)
More than once a week	18(18.8)
**General Knowledge about DM**	
Knowledgeable	208 (93.7)
Not good knowledgeable	14(6.3)
**Knowledge about diabetes self care**	
Knowledgeable	207 (93.2)
Not knowledgeable	15(6.8)
**BMI (body mass index)**	
Normal weight	136 (61.2)
Under weight	13(5.9)
Over weight	60(27.0)
Obese	13 (5.9)

### Knowledge on Diabetes Mellitus

Most of the respondents 208(93.7%) had general knowledge about diabetes. Large proportion 196(88.3%) of the respondents were knowledgeable about the sign and symptoms of diabetes. Majority 207(93.2%) of them were knowledgeable about diabetes self care practices. Substantial proportion 170 (76.6%) reported that regular exercise increases blood glucose level. Less than half 93(41.9%) of them replied that not having breakfast after taking drug increases blood glucose level. Most of them 190(85.6%) answered that non adherence to medication increases blood glucose level.

Regarding knowledge about dietary intake showed that 220(99.1%) had knowledge about honey and simple sugar that have high glycemic index. Among six different types of food which is recommended for patients with diabetes (vegetables, cereals, rice, wheat and its products, potatoes and sweet potatoes, and fruits), 88(39.6%) replied that four of the food had high glycemic index. Less proportion 15(6.8%) of them answered that all the recommended food increase blood glucose level; whereas, only 5(2.3%) of the respondents agreed that all the six recommended food do not increase blood glucose level (Table 2).

### Perceived susceptibility and severity

The perceived susceptibility to diabetes complications indicated that 174(78.4%) of the study respondents had moderate perceived susceptibility and the remaining 39(17.6%) and 9(4.1%) had high and less perceived susceptibility respectively. Regarding perceived severity of diabetes and its related complications, a total of 112(50.5%) respondents had a moderate perceived severity. The other half, 89(40.1%) had high or less 21(9.5%) perceived severity of diabetes and its related complications.

### Perceived barriers and benefits

Among the total respondents there was no high perceived barrier to self care practice. In contrast, majority of the respondents 149(67.1%) had less perceived barrier followed by moderate perceived barriers 63(32.9%) to self care practice to practice further complications related to diabetes mellitus. Furthermore, the perception of respondents about benefit of self care indicated that majority of them 124(55.9%) had high perceived benefit of self care practice. The remaining 98(44.2%) of the respondents had moderate perceived benefit of self care.

### Self efficacy

The respondents self efficacy (confidence) to self care practice related to diabetes revealed that 144(64.9%) had moderate self efficacy and 48(21.6%) had less self efficacy. Only 30 (13.5%) of them had high self efficacy to self care practices related to diabetes mellitus (Table 3).

**Table 3:** Perception of Patients with diabetes on Self Care in Harari; Eastern Ethiopia, 2011.

Variables	Number (%)
**Perceived susceptibility to diabetes complications**	
Less	9 (4.1)
Moderate	174(78.4)
High	39(17.6)
**Perceived severity of Diabetes and its complications**	
Less	21(9.5)
Moderate	112(50.5)
High	89(40.1)
**Perceived barriers to self care**	
Less	149(67.1)
Moderate	73(32.9)
High	0(0.0)
**Perceived benefits of self care**	
Less	0(0.0)
Moderate	98(44.2)
High	124(55.9)
**Self efficacy to perform Self care**	
Less	48(21.6)
Moderate	144(64.9)
High	30(13.5)

### Self care practices

From the total study respondents, 87(39.2%) practiced the recommended self care practices. Before the last three days of the interview, about more than half of the respondents 128(57.7%) followed the recommended dietary intake. Only 69(31.1%) had exercise for thirty minutes per day and 57(25.7%) did not have exercise before the last three days of the interview. Blood glucose monitoring in the last three days before the date of interview showed that 93(41.9%) measured one day and 94(42.3%) did not measure at all. Majority of the respondents 174(78.4%) had taken the prescribed drugs appropriately whereas 10(4.5%) did not take at all.

### Predictors of self care behaviors

According to the result of the multivariate analysis, patients with less frequent information were 0.3 times less likely performed self care [OR-0.3, 95% CI (0.09, 0.79)] than patients with more frequent information about the disease.

Individuals with elementary educational status were around four times more likely to perform self care than unable to read and write individuals [OR-3.9, 95%CI (1.23, 12.18)]. On the other hand, diabetic patients with very high income were 0.2 times less likely to perform self care than with less income [OR-0.2, 95%CI (0.04, 0.75)].

Individuals of high perceived severity of the disease and its complications were 12.3 times more likely to perform self care than less perceived severity [OR-12.3, 95%CI (1.19, 126.25)]. individuals who had moderate perceived barriers to self care were 0.3 times less likely to perform self care than with less perceived barriers [OR-0.3, 95%CI (0.12, 0.64)] (Table 4).

**Table 4:** Predictors of self care behavior among patients with diabetes in Harari, Eastern Ethiopia, 2011.

Variables	High recommended Self care		AOR (95% CI)
	Yes	No	
**Frequency of Information heard**			
High	64	83	1.0
Low	7	21	0.3 (0.09, 0.79)
**Educational status**			
Unable to read and write	30	64	1.00
Below grade six	19	16	3.9 (1.23, 12.18)
Between Grade 6–12	24	36	1.5 (0.62, 3.69)
Above grade 12	14	19	2.2 (0.67,7.04)
**Income per month**			
Low	28	36	1.0
Middle	35	42	1.4 (0.56, 3.33)
High	14	22	1.2 (0.38, 3.42)
Very high	6	23	0.18 (0.04, 0.75)
**Perceived severity of diabetes and its complications**			
Less	5	16	1.0
Moderate	38	74	5.5(0.54,54.76)
High	44	45	12.3 (1.19,126.25)
**Perceived barriers to self care**			
Less	72	77	1.0
Moderate	15	56	0.3 (0.12, 0.64)

## Discussion

This study revealed that type of diabetes was not significantly associated to diabetes self care practice, similar study also showed there were no associations between type of diabetes and self care practices [15]. The mean BMI was 23.8(SD±3.9) and most of them 136(61.2%) were found in the normal weight category and only 13(5.9%) were obese. Our study respondents were less obese compared with other study [16] which may be related with the low socioeconomic status of our study respondents. Further analysis of this study showed that BMI of patients did not have significant statistical influence on diabetes self care behavior. But, similar study done in Jordan patients with diabetes were more likely to be poorly controlled among higher BMI patients [17].

In this study, very few patients 87(39.2%) practiced the recommended self care practices. Among the recommended self care behaviors, drug adherence 174(78.4%) and dietary intake 128(57.7%) were the most practiced recommended self care behaviors than others. On the other hand, regular exercise was the least practiced behavior. This may be unlike drug adherence; the other recommended behaviors may have more perceived and actual barriers. In addition, perceived severity or consequences of not taking prescribed drugs and diets might be higher than the remaining recommended behaviors. It was also supported by similar study in United Arab Emirates [18].

It is known that knowledge is an essential factor for behavior change but it cannot be a sufficient condition to behavior change by its own alone [19]. In this study, Knowledge of the respondents about diabetes was very high accounting 208 (94.7%); however, majority of them did not follow the recommended self care practices. This may be associated to factors such as high perceived barriers of self care, less perceived severity of the disease and its complications, infrequent cues to action, low income and educational status which is supported by the a study done in Nigeria [20]. Similarly, a study conducted in Ethiopia showed that knowledge about diabetes had no significant statistical association with glycemic control [21].

Among six different types of food which is recommended for patients with diabetes such as vegetables, cereals, rice, whites and its products, potatoes and sweet potatoes 88(39.6%) believed that four of the food had high glycemic index such as rice, white, potato and sweat potato's and which is not similar to recommended diabetes diet [22]. A study conducted in Nigeria about knowledge on kind of food a diabetic patient should eat revealed that14.6% opted for whole grains, fresh vegetables and, majority of the respondents (88.5%) stated that avoiding starchy foods is a self-care measure in the prevention/control of diabetes. On self care measures in this study 18(8.1%) of that patients said regular exercise decrease blood glucose levels similarly in Nigeria 7.3% said engaging in regular exercise help to control diabetes [23].

Unlike a study done in Jimma university hospital (Ethiopia) [11], income was one of the factors that affect self care behavior in our study. Generally, high income patients were less adherent to self care than low income patients; this may be due to high income patients may have riskier life style than low income respondents. On the other hand, individuals who had a formal education were more likely to adhere on self care than patients who were unable to read and write. Similar results were found in other studies also [17], [18].

The perceived barriers of respondents showed that people who had moderate perceived barrier to self care were better to perform self care; similar study conducted in Jordan showed that increased perceived barriers to adherence was significantly associated to poor control of diabetes [17]. In line with the assumption of the HBM, patients who had less perceived severity of the disease were more adherent to self care; and according to the model, perceived severity is the strongest predictor to practice [12]. Similarly, a study conducted in Iran showed similar finding [24]. In contrast to the assumptions of the HBM, perceived susceptibility to diabetic complications and perceived benefits of recommended self care were not good predictors of self care on diabetes

It is well known that increasing reach and frequency of appropriate message has an impact on behavior change [25]. In line to that, patients who were with more frequency of information about the disease were three times more likely adhere to self care. This finding is also consistent with a study done in Georgia [26]. In addition, a case-control study conducted in Malaysia showed that patients who followed self efficacy education on self care have brought significant improvement on self care than the control group [27].

Since the data were collected by health professionals working in follow up clinic there might be social desirability bias. Medication adherence, nutritional intake, testing blood glucose and physical activity were obtained by self-report and may be limited by recall bias.

From this study we can conclude that education has a significant effect for patients with diabetes in order to provide own self care practice. This finding also showed that BMI has no significant statistical effect on self care behavior. Patients income also another determining factor for diabetes self care practices as the study showed higher income patients were less to perform self care. Patients with high perceived severity of the disease were more likely to adhere to self care practice, so perceived severity of the disease is helpful for the likely hood adherence of self care. High perceived barriers was also one of the obstacles for patients with diabetes self care adherence. To increase the self care behavior, diabetes messages should focus on severity of diabetes and how to overcome barriers for self care by segmenting the audiences based on income and educational status with increasing the frequency and reach of message on diabetes.
